# Endophytic Bacteria Improve Bio- and Phytoremediation of Heavy Metals

**DOI:** 10.3390/microorganisms12112137

**Published:** 2024-10-24

**Authors:** Ling Liu, Shujing Quan, Liangliang Li, Gao Lei, Shanshan Li, Tao Gong, Zhilong Zhang, Yiliang Hu, Wenling Yang

**Affiliations:** 1 School of Life Sciences, Henan University, Kaifeng 475001, China; lling202203@163.com; 2 Institute of Biology Co., Ltd., Henan Academy of Sciences, Zhengzhou 450008, China

**Keywords:** endophytic bacteria, phytoremediation, heavy metal pollution, combined remediation

## Abstract

Currently, the problem of heavy metal pollution in China is becoming increasingly serious, which poses grave threats to the environment and human health. Owing to the non-biodegradability and toxicity of heavy metals, a more sustainable and ecological approach to remediate heavy metal pollution has always been a focus of attention for environmental researchers. In recent years, many scientists have found that phytoremediation aided by endophytes has high potential to remediate heavy metals owing to its low cost, effectiveness, environmental friendliness, and sustainability compared with physical and chemical methods. Indeed, the mechanism of interaction between endophytes, plants, and heavy metals in the soil is pivotal for plants to tolerate metal toxicity and thrive. In this review, we focus on the mechanism of how endophytic bacteria resist heavy metals, and the direct and indirect mechanisms employed by endophytic bacteria to promote the growth of plants and enhance phytoextraction and phytostabilization. Moreover, we also discuss the application of combinations of endophytic bacteria and plants that have been used to remediate heavy metal pollution. Finally, it is pointed out that although there have been many studies on phytoremediation systems that have been assisted by endophytes, large-scale field trials are important to deliver “real” results to evaluate and improve phytoremediation assisted with microorganisms in polluted natural environments.

## 1. Introduction

The development of the Chinese industrial economy has led to the discharge of many types of heavy metal pollutants into the soil. These heavy metal ions can form chelates with hydroxyl groups, ammonia, and certain molecules in organic matter, which can enable their migration and transformation in the soil [1]. They are easily absorbed by plants, which can cause excessive levels of heavy metals in grains, fruit trees, and other types of crops, thus deleteriously affecting food safety. They then enter the human body through the food chain, which causes various physiological changes and can lead to various acute and chronic diseases, such as chronic poisoning and cancer; thus, they pose a serious threat to the ecological environment and human health [2,3,4]. A variety of soil remediation methods have been utilized to solve the problem of the pollution of soil with heavy metals. The method of physical or chemical remediation is highly effective at remediating the damage, but it will come at the cost of consuming a substantial amount of manpower and material resources or causing secondary pollution. Microbial remediation can change the valence state of heavy metals and reduce their toxicity, but it is difficult to completely remove these pollutants. Phytoremediation is environmentally friendly, but hyperaccumulators grow slowly and take a long time to remediate the soil. The combined remediation of soil contaminated with heavy metals by microbial–plant consortia is green, economical, and can fully leverage the advantages of both microbial and plant functions [5,6]. Thus, it has broad prospects for development. Compared with the microorganisms that live on the plant’s exterior or in the environment, endophytes are better able to coordinate their populations and adapt to their environment and host [7,8]. The symbiotic system between endophytes and plants can enhance the ability of plants to remediate heavy metal pollution [9,10]. Endophytes are currently defined as a group of microorganisms that inhabit various tissues and organs of plants and do not cause any noticeable disease for part or all of their life cycle [11]. The plant biostimulants produced by endophytes can directly and effectively act on the host, and thus regulate the growth of plants under environmental stresses. Endophytes and their metabolites play a dominant role in improving the soil environment, increasing soil enzyme activity, and enhancing the resistance of plants to stress [12,13,14].

## 2. Mechanism of the Resistance of Endophytic Bacteria to Heavy Metals

**Figure 1:**
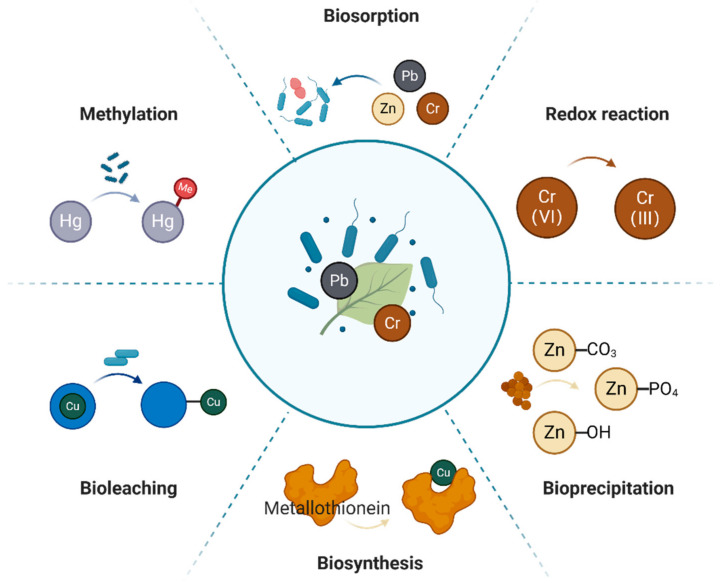
Interaction of endophytic bacteria with heavy metals during the process of bioremediation.

Since endophytes have evolved with the stress of heavy metals for a long time, they have developed various mechanisms of resistance to alleviate the toxicity of heavy metals, and these mechanisms of resistance are coordinated to resist the heavy metals in the environment [15]. The mechanisms used by endophytic bacteria to resist these pollutants include biological adsorption, methylation, redox reactions, bioleaching, bioprecipitation, and biosynthesis [16,17] (Figure 1).

(1) Biosorption. Biosorption is a process in which the functional groups on the cell wall of endophytes bind metal ions to their cell surface, and the cell surface accumulates heavy metals through adsorption, thereby reducing the toxicity of heavy metals to plants. This method can rapidly transfer heavy metal ions into the cells to limit their movement, thereby reducing their toxicity [18]. The bacterial cell wall usually has a negative charge, which enables it to easily react with metals in the outer membrane of the cell, and it is highly effective at forming complexes with dissolved heavy metal ions [19,20]. Raheem et al. [21] found *Bacillus amyloliquefaciens* RWL-1 could adsorb copper (Cu) ions on RWL-1 cell surfaces, which enhanced the seedling biomass of rice (*Oryza sativa*) and increased its levels of antioxidants.
(2) Methylation. Microbial methylation in vivo can effectively reduce the toxic effect of heavy metal ions on organisms. This is particularly true for the methylation of lead (Pb) and mercury (Hg) ions. When the methyl group has a stronger affinity with the divalent mercury ion, it forms methylmercury (MeHg), which weakens the toxicity of Hg. Sulfate-reducing bacteria can catalyze the methylation of Hg. Graham et al. [22] found that some species of *Desulfovibrio* could produce MeHg, and they identified four new Hg methylators (*Desulfovibrio aespoeensis*, *D. alkalitolerans*, *D. psychrotolerans*, and *D. sulfodismutans*). Smith et al. [23] showed that *D. desulfuricans* ND132 could methylate Hg, and Cys73 and Cys93 are the essential amino acids for methylation.
(3) Redox strategies. Heavy metals with different valences, such as arsenic (As), chromium (Cr), and cobalt (Co), are usually found in different valence states in the natural environment. Endophytic bacteria can change the valence of heavy metals through intracellular redox reactions, thereby reducing their toxicity [24]. Xu et al. [25] found that As(III) was oxidized to As(V) after it became attached to the endophytic bacterium W1-2B, which significantly enhanced the strain’s resistance to As(V) and reduced its toxicity.
(4) Bioleaching. Bioleaching is a technology that utilizes microorganisms to recover metals from minerals through the oxidation and dissolution of metal ions [26]. Zhu et al. [27] used a method that combined autotrophic and heterotrophic bacteria to leach the heavy metals zinc (Zn), manganese (Mn), Cu, and cadmium (Cd) from sludge. The auto- and heterotrophic bacteria effectively improved the efficiency of bioleaching, and the main fractions of the heavy metals (Zn, Mn, Cu, and Cd) in the sediments were Fe-Mn oxide, an organic-associated form, and a residual form after bioleaching. The biotoxicity of the sediments decreased greatly.
(5) Bioprecipitation. Plant-growth-promoting rhizosphere bacteria (PGPR) secrete extracellular polymers and other compounds, which directly chelate heavy metals to precipitate or complex extracellular molecules to reduce the toxicity of heavy metals [28].
(6) Biosynthesis. Endophytic bacteria can convert heavy metals into less toxic forms by synthesizing proteins, such as metallothioneins (MTs), glutathione (GSH), and plant lectins (PCs), that are stable to heat and bind to heavy metal ions [29].

## 3. Mechanisms Used by Endophytic Bacteria to Enhance the Phytoremediation of Heavy Metals

**Figure 2:**
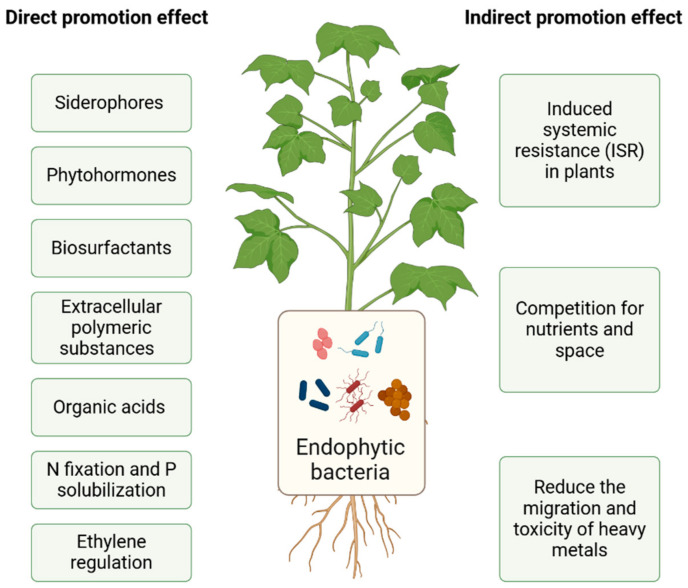
The role of endophytic bacteria in the promotion of plant growth and phytoremediation under heavy metal stress.

The mechanisms used by endophytic bacteria to enhance phytoremediation on heavy metals include changing the activity of the heavy metals, promoting the growth of plants and improving the resistance of plants to heavy metals. The direct mechanism is to directly improve plant nutrition, increase plant biomass, and enhance its resistance to stress by utilizing functions such as the production of siderophores, the biological fixation of nitrogen (N), the solubilization of phosphorus (P), the secretion of organic acids and biosurfactants, the production of extracellular polymeric substances (EPSs), the biosynthesis of specific enzymes, and the regulation of plant hormones [30]. Indirect mechanisms of action primarily include induced systemic resistance (ISR) in the plant, which improves their ability to compete for nutrients and habitat space, changes the form of heavy metals, and reduces their ability to migrate and their toxicity [31] (Figure 2).

### 3.1. Direct Promotion Effect

(1) Secretion of siderophores. Iron in the soil primarily exists in the form of highly insoluble trivalent iron (Fe^3+^) oxides, hydroxides, phosphates, and carbonates, which generally cannot meet the needs of soil microorganisms to reproduce and plants to grow and develop [32]. Siderophores are a class of small molecular compounds that are produced by microorganisms inside the cell and secreted to its exterior. Rhizosphere bacteria produce siderophores in the absence of iron ions in the plant rhizosphere. Siderophores can form complexes with heavy metals, and this process plays an important role in the phytoremediation of heavy metals. Some endophytic bacteria, such as *Pseudomonas*, *Serratia*, and *Streptomyces*, can secrete siderophores. Chen et al. [33] isolated the endophytic bacteria *Serratia nematodiphila* LRE07, *Enterobacter* sp. LRE04, *Enterobacter aerogenes* LRE17, and *Acinetobacter* sp. LSE06 from *Solanum nigrum*. These endophytes can all secrete siderophores, promote plant growth, and improve the ability of plants to remediate Cd pollution in the soil.
(2) N fixation and P solubilization. Nitrogen is an essential nutrient for plant growth. Endophytic azotobacter can directly supply fixed N to plants for absorption and assimilation and promote the growth of their roots [34]. Phosphorus is another essential element that is required for life. It is primarily found in the form of insoluble P in the soil, which is difficult for plants to absorb and utilize. Some endophytic bacteria, such as *Micrococcus luteus* and *Enterobacter cloacae*, are very effective at solubilizing it, which enables the plants to take up this form of P [12].
(3) Secretion of organic acids. Organic acids are acidic organic compounds, which can activate heavy metal ions, change the form of heavy metals, and enhance mineral nutrients. Bacteria in the rhizosphere secrete these compounds to increase the uptake of plant nutrients by inducing root exudates [35].
(4) Secretion of biosurfactants. Biosurfactants are biodegradable low-molecular-weight organic compounds secreted by microorganisms. They include glycolipids, neutral lipid derivatives, and lipoproteins. When the concentration of surfactant exceeds the critical micelle concentration, micelles form in the solution. When heavy metals bind to these micelles, they are transferred to the liquid phase of the soil, which increases their solubility and bioavailability in the soil, thereby favoring phytostabilization [36]. *Pseudomonas aeruginosa*, *Burkholderia* sp., *Paenibacillus* sp., and *Citrobacter freundii* can secrete biosurfactants. They contain a variety of groups with heavy metal complexation and coordination abilities (such as sulfhydryl, carboxyl, hydroxyl, amino, and phosphate groups and bicarbonates), among others, which can be combined with heavy metal ions to form soluble metal–organic complexes; these complexes are more easily absorbed by plants [37,38].
(5) Secretion of EPSs. EPSs are a type of complex polymer that attaches to the surface of bacteria or surrounding microbes. The EPS complexes bind heavy metal ions to form a complex, which reduces the migration of heavy metal ions and therefore reduces their toxicity to plants. Schue et al. [39] showed that *Rhizobium alamii*, an exopolysaccharide-producing bacterium, formed a biofilm on plant roots, which protected the cells of sunflower (*Helianthus annuus*) by preventing Cd from entering them.
(6) Secretion of phytohormones. Phytohormones are compounds that promote the growth, division, and differentiation of plant tissues. Based on their source, these hormones can be divided into endogenous hormones secreted by plants in vitro and exogenous hormones, which primarily include indole-3-acetic acid (IAA), cytokinin (CTK), and gibberellin (GA), among others [40]. Chen et al. [41] inoculated *Lolium perenne* under Cu stress with *Rhizobium* sp. W33, and this strain increased the nutrient and biomass of *L. perenne* and promoted its growth. He et al. [42] found that the endophytic bacteria AR1, AY1, and BG4 isolated from *S. nigrum* can all produce IAA. Moreover, the inoculation of these strains onto rape (*Brassica napus*) contaminated with Cd could promote its growth.
(7) Regulation of ethylene. Some endophytes have ACC deaminase activity, and the precursor ACC of ethylene biosynthesis will be broken down into α-butanoic acid and ammonia molecules, thereby reducing the level of ethylene in plants [43]. Plants can use ammonia molecules as a source of nitrogen, which relieves the pressure caused by adverse conditions of plant growth and development and increases the plant’s absorption of heavy metal ions. The gene *acdS*, which encodes 1-aminocyclopropane-1-carboxylate or ACC-deaminase, is associated with an increase in plant biomass and stress tolerance. The expression of *acdS* decreases the levels of ethylene induced by stress, and the enzyme is abundant in rhizosphere colonizers. *Paraburkholderia dioscoreae* Msb3 that produces ACC-deaminase colonized the leaf sphere of tomato and promoted their growth compared with the controls [44]. Souza et al. [45] found that *Paenibacillus* sp. FeS53 possessed ACC-deaminase activity and promoted the dry shoot biomass of rice and its uptake of nutrients in the presence of excess Fe.

### 3.2. Indirect Promotion Effect

The indirect mechanisms of action primarily include antibiosis, competition for nutrients and space, and changes in the forms of heavy metals. Some endophytes can change the soil conditions, including the content of organic soil matter, activity of soil enzymes, temperature, pH, and level of pollution, among others, and indirectly affect the ability of plants to remediate the soil [46].

(1) Induced systemic resistance (ISR) in plants. Under heavy metal stress, plants will accumulate a large amount of reactive oxygen species (ROS), and the cells will release excessive numbers of free radicals of oxygen, which will destroy the integrity of cell membranes. Endophytic bacteria can induce, activate, and enhance the activities of plant antioxidant enzymes [17], such as peroxidase (POD), superoxide dismutase (SOD), catalase (CAT), ascorbate peroxidase (APX), glutathione reductase (GR), and glutathione peroxidase (GPX) and increase the content of proline, which increases the resistance of plants to heavy metals [32]. The ethylene response factor (ERF) protein plays an important role in the responses to plant stress and developmental regulation. It was reported that JERF3 activated the expression of oxidative stress response genes in transgenic tobacco and enhanced its tolerance to adverse environments [47].
(2) Competition for nutrients and space. The plant endophytes stimulate the plants to secrete specific enzymes, such as hydrolases, along with plant hormones and antibiotics, which regulate the plant rhizosphere microbial community structure, reduce the absorption of heavy metals by plants, and increase plant biomass. The change in the morphology of plant roots caused by endophytes can indirectly promote the growth of plants in environments that are highly polluted with heavy metals [48].
(3) Change the valence state of heavy metals to reduce their ability to migrate and their toxicity. Endophytic bacteria can change the valence state of heavy metals through their mechanisms that limit the metabolism of heavy metals. Insoluble heavy metal ions are transformed into easily soluble ones, which can change them from more toxic to less toxic ionic forms [49]. Endophytic bacteria can bind heavy metal ions through adsorption, exchange, and chelation, which can effectively weaken the migration of heavy metal ions and thus reduce their toxicity to plants [17]. *Serratia marcescens* BacI56 and *Pseudomonas* sp. BacI38 increased the volatilization of Hg in plants by 47.16% and 62.42%, respectively [50].

## 4. Approaches of the Phytoremediation of Heavy Metals Involving Endophytic Bacteria

Endophytic bacteria can promote the growth of plants under heavy metal stress through the fixation of N, the solubilization of P and potassium (K), the secretion of phytohormones, organic acids, chelates, biosurfactants, and siderophores, and the production of specific enzymes, such as ACC-deaminase, chitinase, and antibiotics [51,52,53,54,55,56,57,58]. Govarthanan et al. [59] found that the endophytic bacterium *Paenibacillus* sp. RM isolated from the roots of *Tridax procumbens* could produce IAA, siderophores, ACC-deaminase, secondary metabolites, and biosurfactants and solubilize phosphates and resist Cu (750 mg/L), Zn (500 mg/L), Pb (450 mg/L), and As (400 mg/L). Inoculation with *Acinetobacter baumannii* BacI43, *Bacillus* sp. BacI34, *Enterobacter* sp. BacI14, *Pantoea* sp. BacI23, *Pseudomonas* sp. BacI7, *Pseudomonas* sp. BacI38, and *Serratia marcescens* BacI56 can promote the growth of maize (*Zea mays*) on substrates contaminated with Hg. The strains *A. baumannii* BacI43 and *Bacillus* sp. BacI34 increased the total dry biomass of maize by approximately 47%. The strains *S. marcescens* BacI56 and *Pseudomonas* sp. BacI38 increased the volatilization of Hg by maize by 47.16% and 62.42%, respectively. *Bacillus* sp. BacI34 and *Pantoea* sp. BacI23 promoted the enrichment of Hg in maize tissues [60]. Four endophytic bacteria, *Priestia megaterium* R2.5.2, R3.4.5, and L3.5.1, and *Micrococcus luteus* S3.4.1 were used in a study for the bioremediation of soil contaminated with As. They enhanced fern (*Pteris vittata*) growth and As accumulation [61]. Inoculation with GDB-1 not only significantly increased the biomass, chlorophyll content, and number of root nodules of alder (*Alnus firma)* seedlings but also enhanced the accumulation of heavy metals, including As, Cu, Pb, Ni, and Zn [62]. The inoculation of maize seeds with *Agrococus tereus* significantly increased the shoot length, root length, leaf width, plant height, fresh weight, moisture content, proline, and activities of POD and SOD, enhanced its absorption of Cu, Mn, Ni, Na, Cr, Fe, Ca, Mg, and K, and reduced the toxicity of Zn and Ni. Therefore, the strain of *A. tereus* could potentially be utilized for the detoxification of heavy metals [63]. *Sphingomonas* sp. ZYG-4 isolated from the roots of *Ageratina adenophora* could secrete IAA, solubilize phosphate, and regulate root ethylene levels and increase the accumulation of Pb in the buds and the root biomass [64].

Endophytic bacteria can improve the efficiency of phytoremediation on soil contaminated with heavy metals by changing the form and bioavailability of heavy metals and the state of roots. Wang et al. [65] found that the expression of the Zn transporters SaIRT1 and SaNramp1 in the roots increased after inoculation with the endophytic bacterium *Pseudomonas fluorescens* Sasm05, and the endophytic bacteria promoted the development of *Sedum alfredii* roots and their uptake of heavy metals. Wang et al. [66] suggested that after inoculation with *B. cereus* BL4, there were increases in the bioavailability of Cd in the soil, the activity of soil enzymes, such as sucrase, urease, alkaline phosphatase, dehydrogenase, FDA hydrolase, and CAT and increases in the richness and diversity of soil bacteria. Moreover, inoculation with *B. cereus* BL4 increased the plant height, fresh weight, chlorophyll content, photosynthetic rate, and root activity, while it decreased the content of malondialdehyde (MDA). This indicated that the cytotoxicity of Cd was reduced and the phytoremediation of *Miscanthus floridulus* against Cd was enhanced. Feng et al. [67] concluded that the endophytic bacterium LSE03 was effective at leaching and immobilizing Cu^2+^ and Zn^2+^. The removal rates of available Cu and available Zn were 97.92% and 96.39%, respectively, and the soil fertility increased significantly.

In addition, endophytic bacteria can improve the nutritional status of their host plants by secreting auxin, alleviating ethylene stress, increasing the uptake of N, P, and Fe, and enhancing the activity of their antioxidant system to promote their growth [68]. Bilal et al. [69] inoculated *Penicillium funiculosum* LHL06 on soybean (*Glycine max*) roots under combined heavy metal (Ni, Cu, Pb, Cr, and Al) stress and found that LHL06 significantly upregulated the antioxidant system of soybean by enhancing the activities of reduced glutathione (GSH), CAT, POD, and SOD to counteract oxidative stress. Zong et al. [70] suggested that *Bacillus altitudinis* WR10 is a Cu-resistant strain that could reduce its toxicity to wheat (*Triticum aestivum*) by enhancing the expression and activity of POD, increasing the level of GSH, removing active oxygen species in vivo, and downregulating the expression of glutathione S-transferase (GST).

Genetic engineering can substantially improve the remediation efficiency of the phytoremediation of heavy metal pollution enhanced by endophytes. Gu et al. [71] isolated the genera *Agrobacterium* and *Bacillus* producing high levels of IAA from the roots of Chinese brake fern (*P. vittata*) with different concentrations of As. In addition, they found that the genes *arsB* and *ACR3(2)* were transferred among the strains, which indicated that *arsB* and *ACR3(2)* might be heavy-metal-As-resistant genes. Qiu et al. [72] introduced the bifunctional GSH synthase gene *gcsgs* into the endophytic *Enterobacteriaceae* genus CBSB1 and named it CBSB1-GCSGS. The inoculation of CBSB1-GCSGS and CBSB1 in soil that was contaminated with Cd and Pb significantly increased the extraction of Cd and Pb from *Brassica juncea* seedlings, with increases in aboveground length, fresh weight, and dry weight of 67%, 123%, and 160%, respectively.

Moreover, the inoculation with an endophytic consortium significantly enhanced the remediation efficiency of phytoremediation under heavy metal stress. The inoculation with *Phialophora mustea* Pr27 and *Leptodontidium* sp. Me07 improved mineral nutrition of *Noccaea caerulescens* and increased the extraction of Zn (30%) and Cd (90%), respectively [9]. Ouyang et al. [73] inoculated alfalfa (*Medicago sativa*) with *Klebsiella variicola* Y38 and *Serratia surfactantfaciens* Y15 in soil contaminated with Cd and found that the inoculated strains enhanced its growth and antioxidant capacity, altered the morphology of Cd in the soil, and increased the bioaccumulation and transfer coefficient of Cd. Under the dual stress of Cu^2+^ and Zn^2+^ at 400 mg/kg, a double inoculation with *Sinorhizobium meliloti* CCNWSX0020 and *Agrobacterium tumefaciens* CCNWGS0286 significantly increased the number of nodules, root length, and biomass of *M. lupulina* by enhancing its antioxidant activity and increasing the total uptake of Cu compared with a single bacterial inoculation by 39.1% and 47.5%, respectively, which resulted in 35.4% and 44.2% increases in the total absorption of Zn, respectively [74]. Shilpee et al. [75] found that inoculation with the endophytic bacterial strains *Bacillus aryabhattai* AS03 and *Rhizobium pusense* AS05 in soil contaminated with Cr(VI) promoted an increase in the stem length, root length, nodule number and leghemoglobin content of *Macrotyloma uniflorum* var. Madhu, while the contents of ROS and activities of the antioxidant enzymes decreased. Moreover, the transpiration rate, total photosynthetic rate, and intracellular concentrations of the CO_2_ of the plants increased significantly after a double inoculation. Ashraf et al. [76] inoculated the endophytes *Enterobacter* HU38, *Microbacterium arborescens* HU33, and *Pantoea stewartii* AsI11 in soil contaminated with Cr and identified a significant increase in the root length, stem height, chlorophyll content, and total biomass of *Leptochloa fusca*.

Combined remediation has also been highly effective at remediating heavy metal pollution in the soil. Sabir et al. [77] found that the inoculation of *Enterobacter* sp. MN17 and biochar to soil contaminated with Cd could significantly reduce the MDA content and activities of antioxidant enzymes, such as CAT, GPX, GST, and SOD, of *Brassica napus*. The combined application of the endophytic bacterium MN17 and biochar reduced the concentration of Cd in the soil by 45.6%, thereby reducing its uptake in the root and aboveground tissues of *B. napus*. Naveed et al. [78] showed that mixing *Enterobacter* sp. MN17, biochar, and gravel sand into soil contaminated with Cd resulted in an increase in plant height (47%), aboveground dry weight (42%), and root weight (57%) of pea (*Pisum sativum*) and a decrease in the uptake of Cd by the roots and migration to the aboveground tissues. In addition, the components of biochemical indicators, such as protein, fat, fiber, and ash, in the seeds increased significantly by up to 41%. Nafees et al. [79] studied the effect of the combined treatment of biogas slurry (BGS) and *Burkholderia phytofirmans* PSJN in soil contaminated with Cr and found that the combined treatment could effectively stabilize Cr in the soil, minimize absorption in the belowground parts and roots of *B. napus*, reduce the activities of glutathione peroxidase (GSH-Px) in the soil to normal levels, and promote the growth of this plant in the contaminated soil. Shahzad et al. [80] found that the treatment of wheat with *Bacillus mycoides* and rock phosphate increased the shoot length, leaf width, protein, sugar content, and Mn, Na, and K contents, while it decreased the antioxidant enzymes SOD and POD in the contaminated soil and inhibited the accumulation of Ni and Zn by 63%.

As shown in Table 1, substantial progress has been made on the remediation of heavy metal pollution by endophytic bacteria throughout the world during the past decade.

## 5. Conclusions and Future Perspectives

The use of endophytic bacteria in phytoremediation has a substantial potential to remediate heavy metals owing to its cost-effectiveness, efficiency, environmental friendliness, and sustainability. Endophytic bacteria resist heavy metals through biosorption, bioleaching, bioprecipitation, biosynthesis, and biotransformation and utilize direct and indirect mechanisms to promote plant growth and enhance phytoextraction and phytostabilization. The application of endophytic bacteria in agriculture and environmental sustainability is a constantly developing research field. The latest advances in biotechnology and bioinformatics tools, such as RNA interference (RNAi), metabolomics, and next-generation sequencing systems, have made it possible to study endophytic bacteria at the molecular level. Increasing numbers of endophytic bacteria with multiple functions in different severe habitats have been discovered and applied in environmental remediation, which has improved crop productivity. The active substances produced by endophytic bacteria are gradually being developed and commercialized. However, large-scale field trials are important to deliver “real” results to evaluate and improve phytoremediation assisted by microbes in polluted natural environments. The actual polluted soil often contains other pollutants besides heavy metals. The combination of endophytic bacteria and plants for heavy metal remediation is easily affected by soil texture, soil nutrients, temperature, and competition from local microorganisms. Therefore, the practical application of this method in natural environments is still difficult. Endophytic bacteria are susceptible to severe environmental conditions, which makes it challenging to stabilize their growth-promoting effects. Using such techniques to modify endophytic bacteria opens a new chapter in the field of environmental remediation. Genetic engineering technology may help to enhance the potential of endophytic bacteria and accelerate their application in the phytoremediation of soil contaminated with heavy metals. Moreover, nanoparticles loaded with endophytic bacteria not only enhance their growth-promoting efficiency but also facilitate better adaptation to the adverse external environment. Therefore, endophytes provide a valuable platform for improving bioremediation models for heavy metal pollutants, along with other approaches, to manage environmental pollution.

## Figures and Tables

**Table 1:** Plant-endophytic bacteria combinations in various heavy metal phytoremediation studies.

Endophytic Bacteria	Host Plants	Effect/Mechanism	Heavy Metal(s)	References
*Phialophora mustea* Pr27; *Leptodontidium* sp. Me07	*Noccaea caerulescens*	Improvement in plant mineral nutrition; Uptake of Zn (30%) and Cd (90%).	Zn, Cd	[9]
*Bacillus amyloliquefaciens* RWL-1	*Rice*	Enhancement in seedling biomass and antioxidant levels (POD, PPO, and GHS); Increase of seedling biomass.	Cu	[21]
FeS53	*Rice*	Promotion of plant growth and nutrient absorption; Alleviation of the effect of Cd stress.	Fe	[45]
*Pseudomonas putida* RE02	*Trifolium repens*	Promotion of IAA; P solubilization; Increase of plant biomass.	Cd	[52]
*Cupriavidus* sp. HXC-8	*Mimosa pudica*	Improvement in plant length, biomass, chlorophyll contents, and GSH; Alleviation of the effect of Cd stress.	Cu	[53]
*Serratia* sp. AI001; *Klebsiella* sp. AI002	*Solanum nigrum*	Improvement in the chlorophyll content, plant length, and biomass; Induction of Cd translocation.	Cd	[54]
*Mesorhizobium loti* HZ76	*Robinia pseudoacacia*	N fixation and production of ACC-deaminase; Improvement in shoot biomass; Decrease in the concentrations of heavy metals (Zn, Pb, Cd) in the roots.	Zn, Pb, and Cd	[55]
*Pseudomonas lurida* EOO26	*Helianthus annuus*	Increase in the length and dry weight of plant root and shoot.	Cu	[57]
*Buttiauxella* sp. SaSR13	*Sedum alfredii*	Production of IAA and decrease in the concentrations of superoxide anion (O_2_^−^) in plants.	Cd	[58]
*Paenibacillus* sp. RM	*Tridax procumbens*	Production of secondary metabolites, IAA, siderophores, ACC-deaminase, and biosurfactants; Solubilization of phosphate; Resistance to Cu (750 mg/L), Zn (500 mg/L), Pb (450 mg/L), As (400 mg/L).	Cu, Zn, Pb, and As	[59]
*7 different endophytic bacteria*	*maize*	Increase in total dry biomass of plants and Hg volatilization.	Hg	[60]
*Priestia megaterium* R2.5.2, *Micrococcus luteus* S3.4.1, *P. megaterium* R3.4.5, and *P. megaterium* L3.5.1)	*Pteris vittata*	Enhancement in biomass; As accumulation.	As	[61]
*Bacillus thuringiensis* GDB-1	*Alnus firma*	Improvement in plant growth and ACC-deaminase activity; Production of IAA, siderophores, and P; Accumulation of heavy metals (including As, Cu, Pb, Ni, and Zn).	As, Cu, Pb, Ni, and Zn	[62]
*Sphingomonas* sp. ZYG-4	*Ageratina adenophora*	Secretion of IAA; Solubilization of phosphate; Regulation of root ethylene levels; Increase in root biomass; Pb accumulation.	Pb	[64]
*Pseudomonas fluorescens* Sasm05	*Sedum alfredii*	Increase in chlorophyll content, plant biomass, and the expression of Zn transporters SaIRT1 and SaNramp1 in the roots.	Zn	[65]
*Bacillus cereus* BL4	*Miscanthus floridulus*	Increase in fresh weight, plant height, chlorophyll content, photosynthetic rate, and root activity; Decrease in MDA content; Increase in the bioavailability of Cd in the soil and the activity of soil enzymes (sucrase, urease, alkaline phosphatase, dehydrogenase, FDA hydrolase, and CAT); increase in the richness and diversity of soil bacteria.	Cd	[66]
*Penicillium funiculosum* LHL06	*Glycine max*	Enhancement in the activities of antioxidant systems (GSH, CAT, POD, and SOD); Alleviation of oxidative stress.	Ni, Cu, Pb, Cr, and Al	[69]
*Bacillus altitudinis* WR10	*Triticum aestivum*	Enhancement in the expression and activity of POD; Down-regulation of GST; Increase in GSH level.	Cu	[70]
*Enterobacteriaceae genus* CBSB1	*Brassica juncea*	Increase in aboveground length, fresh weight, and dry weight; Extraction of Cd and Pb.	Cd, Pb	[72]
*Klebsiella variicola* Y38; *Serratia surfactantfaciens* Y15	*Medicago sativa*	Enhancement in plant growth and antioxidant capacity; Alteration of Cd morphology in soil; Cd bioaccumulation.	Cd	[73]
*Sinorhizobium meliloti* CCNWSX0020; *Agrobacterium tumefaciens* CCNWGS0286	*Medicago lupulina*	Increase in root length and dry weight; Cu uptake.	Cu	[74]
*Bacillus aryabhattai* AS03; *Rhizobium pusense* AS05	*Macrotyloma uniflorum*	Increase in stem length, root length, nodule number, and leghemoglobin content; Decrease in the contents of ROS and activities of antioxidant enzymes.	Cr	[75]
*Enterobacter* HU38, *Microbacterium arborescens* HU33, and *Pantoea stewartii* AsI11	*Leptochloa fusca*	Increase in root length, stem height, chlorophyll content, and total biomass.	Cr	[76]
*Enterobacter* sp. MN17	*Pisum sativum*	Enhancement in plant health; Alleviation of the toxic effects of Cd on plants.	Cd	[78]
*Burkholderia phytofirmans* PsJN	*Brassica napus*	Promotion of plant growth; Reduction in the GSH-Px activity in soil; Stabilization of Cr in the soil.	Cr	[79]

## Data Availability

No new data were created or analyzed in this study. The original contributions presented in the study are included in the article, further inquiries can be directed to the corresponding author.
